# Factors Associated With In-Hospital Mortality in Mycosis Fungoides Patients: A Multivariable Analysis

**DOI:** 10.7759/cureus.28043

**Published:** 2022-08-15

**Authors:** Amber Loren O King, Victor Lee, Fatima N Mirza, Vikram Jairam, Daniel X Yang, James B Yu, Henry S Park, Michael Girardi, Lynn D Wilson, Yi An

**Affiliations:** 1 Department of Dermatology, Yale School of Medicine, New Haven, USA; 2 Department of Therapeutic Radiology, Yale School of Medicine, New Haven, USA; 3 Department of Dermatology, Brown University, Providence, USA; 4 Department of Therapeutic Radiology, Columbia University, New York City, USA

**Keywords:** mortality, sepsis, sezary syndrome, neds, cutaneous t-cell lymphoma, ctcl, mycosis fungoides

## Abstract

Background

Mycosis fungoides (MF) is the most common form of cutaneous T-cell lymphoma (CTCL). Although it often has an indolent course, it can progress to more aggressive CTCL forms. There is sparse data in current literature describing specific clinical factors associated with in-hospital mortality in mycosis fungoides patients. An understanding of patients at greatest risk for in-hospital mortality can aid in developing recommendations for prophylaxis and empirical management.

Aim

We aim to characterize factors associated with in-hospital mortality in MF patients.

Materials and methods

The Nationwide Emergency Department Sample (NEDS) was queried for MF cases from 2006 to 2015. Baseline demographic and hospital characteristics were stratified based on survival outcomes. Multivariable logistic regression was used to identify factors associated with in-hospital mortality.

Results

A total of 57,665 patients with MF presenting to the ED between 2006 and 2015 were identified. Sézary syndrome, sepsis, and advanced age were associated with MF in-hospital mortality, while female sex was inversely associated. There was a downtrend in in-hospital mortality among MF patients presenting to the ED from 2006 to 2015.

Conclusions

Our study highlights factors crucial for risk-stratification for hospitalized MF patients.

## Introduction

Cutaneous T-cell lymphoma (CTCL) is a group of non-Hodgkin lymphomas that affect the skin. Mycosis fungoides (MF) is the most frequent form of CTCL. MF has an estimated prevalence of up to 6.6 per 100,000 people in the US. Although MF typically has an indolent course, it can progress to more aggressive forms of CTCL, including involving the blood and lymph nodes as in Sézary syndrome. The median age of diagnosis is approximately 58 years [1]. However, it is estimated that patients live with the early-stage disease for about 18 years [1]. Although promising novel therapies in active development are grounded on an improved understanding of CTCL pathogenesis, five-year survival in MF patients varies largely by stage. Currently, available treatments are often not curative.

Epidemiological analyses have focused on characterizing demographic data, prevalence, comorbidities, secondary malignancies, and survival rates. However, there remains a paucity of analyses focusing on specific factors associated with mortality for MF patients admitted to the hospital [2]. An understanding of patients at greatest risk for in-hospital mortality can aid in developing recommendations for prophylaxis and empirical management. In addition, characterizing mortality-associated factors can aid in the identification of higher-risk patients and can prioritize close monitoring of particular clinical parameters. To this end, our study analyzes the various factors associated with in-hospital mortality for MF patients who present to the ED in the United States and are admitted for hospitalization.

## Materials and methods

This retrospective analysis utilizes the Nationwide Emergency Department Sample (NEDS) published by the Healthcare Cost and Utilization Project, Agency for Healthcare Research and Quality (HCUP). NEDS encompasses more than 950 hospitals in 34 states and is the largest all-payer ED database in the United States. NEDS contains approximately 25-35 million yearly ED visits. Each visit is assigned a weighted value during the sampling process by HCUP to generate a national estimate. All diagnoses reported in NEDS were based on the International Classification of Diseases, Ninth Revision, Clinical Modification (ICD-9-CM) system until September 30, 2015. The Tenth Revision (ICD-10-CM) was used thereafter. This study was granted an Institutional Review Board exemption by the Yale Human Investigations Committee. Informed consent was waived because the study was retrospective, and data were deidentified. NEDS was queried from January 2006 to December 2015 for all patients with any listed primary or secondary diagnosis of MF (ICD-9-CM: 202.1x and ICD-10-CM: C84.0x). Hospital admissions were characterized by available demographic factors (age, sex, race), socioeconomic factors (insurance type, income), clinical factors (any listed diagnosis of sepsis, anemia, neutropenia, and Sézary syndrome), hospital characteristics (region, location, size, teaching status), and inpatient mortality rate.

Demographic, socioeconomic factors, and hospital characteristics were compared between MF patients based on mortality status during their visit using the Chi-squared test for categorical variables and the Mann-Whitney U or analysis of variance tests for continuous variables. Multivariable logistic regression was used to identify factors associated with in-hospital mortality. Clinical variables chosen for analysis were determined a priori based on implicated disease and treatment-related factors. Neutropenia was excluded from the multivariable analysis due to its clinical association with sepsis. This correlation was confirmed using Chi-squared analysis. Weighted frequencies were used to create national estimates for all data analyses. A trend analysis was performed by comparing year categories in a multivariate analysis. Hypothesis testing was two-sided, and P <0.05 was used to indicate statistical significance for all comparisons. Data analysis was performed using Stata, version 13.1 (StataCorp LP, TX, USA).

## Results

Between 2006 and 2015, there was a weighted total of 57,665 ED visits for MF patients, of which 4,143 (7.2%) died during their associated hospitalization. The characteristics of this cohort are described in Table 1. The mean age of the overall cohort of MF patients was 61.1 ± 20.3 (SD). The majority of visits were male (55.9%), used Medicare (53.9%), were admitted to a larger hospital (58.4%), and presented from 2011 to 2015 (56.9%). The median length of stay was 7.8 days. The median charge per stay was $34,004.50 (interquartile range $17,712 to $67,719).

**Table 1:** Sociodemographic, clinical, and hospital-related characteristics of mycosis fungoides patients in the ED from 2006 to 2015.

Variable	Weighted frequency (%)			P-value
	All Patients	Did not die	Died	
Total Number, N (weighted %)	57665 (100.0)	53522 (92.8)	4143 (7.2)	
Age (mean, years)	61.1	60.7	66.1	<0.001
Age category (years)				<0.001
<65	28496 (49.4)	26746 (50.0)	1750 (42.2)	
≥65	29169 (50.6)	26776 (50.0)	2393 (57.8)	
Sex				<0.001
Male	32204 (55.9)	29627 (55.4)	2577 (62.2)	
Female	25445 (44.1)	23879 (44.6)	1566 (37.8)	
Median Household Income				0.332
$1-$41,999	13791 (24.6)	12778 (24.5)	1013 (25.3)	
$42,000 - $51,999	13288 (23.7)	12409 (23.8)	879 (21.9)	
$52,000 - $67,999	13700 (24.4)	12769 (24.5)	932 (23.3)	
≥$68,000	15303 (27.3)	14121 (27.1)	1183 (29.5)	
Primary Payer				0.147
Medicare	31045 (53.9)	28664 (53.6)	2381 (57.6)	
Medicaid	7935 (13.8)	7345 (13.7)	590 (14.3)	
Private	15336 (26.6)	14400 (27.0)	935 (22.6)	
Self-Pay	1580 (2.7)	1477 (2.8)	103 (2.5)	
No charge	216 (0.4)	202 (0.4)	14 (0.3)	
Other	1447 (2.5)	1340 (2.5)	107 (2.6)	
Hospital Region				0.003
Midwest	11863 (20.6)	11209 (20.9)	654 (15.8)	
Northeast	13349 (23.1)	12253 (22.9)	1096 (26.4)	
South	20826 (36.1)	19263 (36.0)	1563 (37.7)	
West	11627 (20.2)	10797 (20.2)	830 (20.0)	
Hospital Teaching Status				0.022
Non-Teaching	23998 (41.6)	22431 (41.9)	1568 (37.8)	
Teaching	33666 (58.4)	31091 (58.1)	2575 (62.2)	
Year Category				0.002
2006-2010	24844 (43.1)	22843 (42.7)	2001 (48.3)	
2011-2015	32821 (56.9)	30678 (57.3)	2142 (51.7)	
Season				0.557
Winter	11936 (24.6)	11128 (24.7)	808 (23.1)	
Spring	12503 (25.8)	11532 (25.6)	971 (27.8)	
Summer	12911 (26.6)	11996 (26.7)	916 (26.2)	
Fall	11137 (23.0)	10336 (23.0)	801 (22.9)	
Sézary Syndrome				<0.001
No Sézary Syndrome	56666 (98.3)	52667 (98.4)	3999 (96.5)	
Sézary Syndrome	999 (1.7)	854 (1.6)	144 (3.5)	
Sepsis				<0.001
No Sepsis	48703 (84.5)	46920 (87.7)	1784 (43.1)	
Sepsis	8961 (15.5)	6602 (12.3)	2359 (56.9)	
Anemia				<0.001
No anemia	37888 (65.7)	35533 (66.4)	2355 (56.8)	
Anemia	19777 (34.3)	17988 (33.6)	1788 (43.2)	
Neutropenia				0.239
No Neutropenia	52041 (90.2)	48254 (90.2)	3787 (91.4)	
Neutropenia	5624 (9.8)	5268 (9.8)	356 (8.6)	
Disposition				
Admitted	44632 (77.4)	40600 (75.9)	4032 (97.3)	<0.001
Discharged	11261 (19.5)	11261 (21.0)	0 (0.0)	<0.001
Mean Length of Stay (days)	7.8	7.4	12.1	<0.001
Median Charges (dollars)	34004.50	32337.00	68703.39	<0.001

Baseline characteristics of patients grouped by mortality status are outlined in Table 1. Among the 4,143 visits that resulted in death, several comorbidities were listed as a primary or secondary diagnosis. A total of 144 (3.5%) had Sézary syndrome, 2,359 (56.9%) had sepsis, 1,788 (43.2%) had anemia, and 356 (8.6%) had neutropenia.

On unadjusted univariate analysis (all p <0.05), patients that died are more likely to be male (62.2%), be over the age of 65 (57.8%), present to a teaching hospital (62.2%), have a longer mean length of stay (12.1 vs. 7.4 days, p<0.001), and had higher median charge ($68,703.39 vs. $32,337, p<0.001). In addition, deceased patients are more likely to have Sézary syndrome (3.5%), sepsis (56.9%), and anemia (43.2%). On multivariable regression, multiple patient and hospital-related factors were associated with increased in-hospital mortality among ED visits for MF patients after correcting for several demographic variables, including race and sex (Table 2). Patients who were over 65 years old (OR: 1.78; 95% CI: 1.40-2.25), on Medicaid (OR: 1.20; 95% CI: 0.90-1.61), presented to a hospital in the Northeast (OR: 1.42; 95% CI: 1.11-1.82) or South (OR: 1.41; 95% CI: 1.11-1.79), had Sézary Syndrome (OR: 1.80; 95% CI: 1.05-3.06), or had sepsis (OR: 9.42; 95% CI: 8.00-11.08) were more likely to die in the hospital. Patients that were female (OR: 0.74; 95% CI: 0.64-0.88) or presented in 2011-2015 vs. 2006-2010 (OR: 0.70; 95% CI: 0.59-0.84) were less likely to die in the hospital (Table 2).

**Table 2:** Multivariate analysis examining factors associated with mortality for mycosis fungoides ED visits from 2006 to 2015.

Variable	OR	95% CI	P-value
Age category (years)			
<65 (ref)			
≥65	1.78	1.40-2.25	<0.001
Sex			
Male (ref)			
Female	0.74	0.62-0.88	<0.001
Median Household Income			
$1-$41,999 (ref)			
$42,000 - $51,999	0.90	0.70-1.17	0.435
$52,000 - $67,999	0.96	0.75-1.22	0.723
≥$68,000	1.09	0.86-1.37	0.491
Primary Payer			
Medicare (ref)			
Medicaid	1.20	0.90-1.61	0.214
Private	0.96	0.74-1.25	0.778
Self-Pay	1.49	0.89-2.49	0.129
No charge	1.14	0.53-2.44	0.740
Other	1.47	0.78-2.75	0.230
Hospital Region			
Midwest (ref)			
Northeast	1.42	1.11-1.82	0.005
South	1.41	1.11-1.79	0.005
West	1.24	0.96-1.61	0.098
Hospital Teaching Status			
Non-teaching Hospital (ref)			
Teaching Hospital	1.15	0.97-1.37	0.112
Year Category			
2006-2010 (ref)			
2011-2015	0.70	0.59-0.84	<0.001
Season			
Winter (Jan-Mar) (ref)			
Spring (Apr-June)	1.08	0.86-1.36	0.494
Summer (July-Sept)	0.98	0.78-1.24	0.871
Fall (Oct-Dec)	0.99	0.79-1.25	0.936
Sézary Syndrome			
No Sézary Syndrome (ref)			
Sézary Syndrome	1.80	1.05-3.06	0.031
Sepsis			
No Sepsis (ref)			
Sepsis	9.42	8.00-11.08	<0.001
Anemia			
No Anemia (ref)			
Anemia	1.12	0.93-1.34	0.222

## Discussion

Several large-scale epidemiologic studies support the observed association between sepsis and in-hospital mortality in MF patients. Staphylococcus aureus infections are the most common cause of disease-related death in CTCL. While skin colonization is common and responsive to conservative measures such as bleach baths, [3] bacterial sepsis is the most common infectious complication in CTCL. Infectious complications have been reported to be involved in as much as 50% of CTCL deaths [4]. MF skin lesions and procedural interventions such as skin biopsies and indwelling catheters mechanically disrupt the skin barrier, providing potential portals for infections [4].

Immunosuppression due to the disease and treatment-related factors further increases infection risk for CTCL patients. Inherent to CTCL disease development and progression, loss of T-cell receptor complexity and immune dysregulation causes increased susceptibility to infectious agents and the immunosuppression phenotype in CTCL [5,6]. Treatment-related factors such as off-target effects of systemic therapies and invasive modes of administration further heighten infection risk for CTCL patients. In the United States, synthetic retinoid bexarotene, photopheresis, and histone deacetylase (HDAC) inhibitors are most commonly prescribed as first-line treatment for MF/SS [7]. Although bexarotene is generally well-tolerated with reversible side effects, leukopenia was reported in 11% of patients enrolled in multinational trials [8]. In cohorts of patients chronically treated with photopheresis, it was observed that most patients developed iron deficiency anemia [9,10]. Similarly, clinically observed toxicities with IV-administered HDAC inhibitor romidepsin include neutropenia, lymphopenia, and sepsis [11,12]. In vivo and in vitro studies have shown that romidepsin broadly dampens the innate immune response by inhibiting natural killer (NK) cell-mediated cytotoxicity and activation of dendritic cells, thus increasing risk for infection [13].

The financial and demographic factors are found to be associated with inpatient mortality support and offer a broader context for findings in other retrospective analyses. The association of advanced age with mortality can be largely attributed to more advanced disease, increased likelihood of comorbidities, and diminished immune response to fight infection [14,15]. The association of higher median costs and increased length of stay with mortality can be attributed to more complex, higher acuity visits requiring greater level of care and resources [16]. Additionally, the association of Medicaid insurance status with mortality during an in-hospital visit aligns with social disparities in CTCL patient outcomes brought to light by other nationwide retrospective analyses [17]. It has been previously noted that patients on Medicaid presented at later stages than those with private insurance. Potential structural or institutional barriers to timely diagnosis and factors associated with disparities in CTCL patient management warrants further exploration. 

Interestingly, it was seen that patients who presented in 2011-2015 were less likely to die in the hospital when compared to those presenting in 2006-2010, suggestive of continued improvement in the management of MF-related complications within the past decade [18,19]. Decreased MF in-hospital mortality rates in recent years may reflect enhanced empirical infection management and prophylaxis protocols [20]. Another possibility is that the improved survival in more recent years may reflect a clearer consensus on treatment guidelines and the introduction of new targeted treatments that have a less suppressive effect on the immune response. Although phototherapy has been widely used in CTCL for decades, the United States Cutaneous Lymphoma Consortium only recently established standardized guidelines [21]. Other targeted therapies, such as toll-like receptor agonists, immune checkpoint inhibitors, and monoclonal antibodies, have been under active clinical investigation for broader use in CTCL [22-25]. In future studies, the effects of introducing these novel targeted therapies on largescale CTCL patient outcomes remain to be seen.

By nature of nationwide retrospective analyses spanning several years of patient data, data from this investigation must be interpreted with caution. Our study has several limitations, including a lack of availability of other critical mortality-risk factors in the database, potential inter-institutional nuances in data recording, and year-to-year changes in classifications and coding. For the purposes of our analyses, the primary diagnosis was assumed to be the reason for presenting to the ED. It is possible that MF patients present to ED for reasons that are not MF-related. In addition, while unique visits are documented in the database, it is impossible to discern whether they represent duplicate patients. Importantly, information regarding treatment type was not available in the NEDS database for inclusion in our multivariate analysis. However, given the potential side effects of therapies that can lead to increased risk for systemic infections, the type of treatment is demonstrably an essential factor to consider for assessing the risk of mortality in CTCL. Furthermore, retrospective analyses have previously demonstrated that the advanced stage is among the many risk factors for bacteremia [4]. Unfortunately, information regarding the disease stage was unavailable in the NEDS database; however, Sézary syndrome was included as a factor in our multivariate analysis to assess the association of advanced disease with in-hospital mortality in CTCL.

## Conclusions

Nevertheless, despite these limitations, our study highlights factors crucial for the risk-stratification of hospitalized MF patients. An understanding of patients at greater risk for in-hospital mortality can aid in the development of recommendations for prophylaxis and empirical management. Given that CTCL therapies are often palliative, characterizing factors and clinical parameters of concern upon presentation to the ED is crucial for minimizing the burden of disease, potential side effects of systemic therapies, and ultimately improving patient outcomes.
